# Phosphorylation Induced Conformational Transitions in DNA Polymerase *β*

**DOI:** 10.3389/fmolb.2022.900771

**Published:** 2022-06-13

**Authors:** Amit Srivastava, Haitham Idriss, Kamal Taha, Sungmun Lee, Dirar Homouz

**Affiliations:** ^1^ Department of Physics, Khalifa University of Science and Technology, Abu Dhabi, United Arab Emirates; ^2^ Palestinian Neuroscience Initiative, AlQuds University, Jerusalem, Palestine; ^3^ School of Public Health, Imperial College of Science, Technology and Medicine, London, United Kingdom; ^4^ Department of Electrical Engineering and Computer Science, Khalifa University of Science and Technology, Abu Dhabi, United Arab Emirates; ^5^ Biomedical Engineering Department, Khalifa University of Science and Technology, Abu Dhabi, United Arab Emirates; ^6^ Department of Physics, University of Houston, Houston, TX, United States; ^7^ Center for Theoretical Biological Physics, Rice University, Houston, TX, United States

**Keywords:** DNA polymerase β, MD simulation, phosphorylation, conformational changes, principal component analysis

## Abstract

DNA polymerase *β* (pol *β*) is a member of the X- family of DNA polymerases that catalyze the distributive addition of nucleoside triphosphates during base excision DNA repair. Previous studies showed that the enzyme was phosphorylated *in vitro* with PKC at two serines (44 and 55), causing loss of DNA polymerase activity but not DNA binding. In this work, we have investigated the phosphorylation-induced conformational changes in DNA polymerase *β* in the presence of Mg ions. We report a comprehensive atomic resolution study of wild type and phosphorylated DNA polymerase using molecular dynamics (MD) simulations. The results are examined *via* novel methods of internal dynamics and energetics analysis to reveal the underlying mechanism of conformational transitions observed in DNA pol *β*. The results show drastic conformational changes in the structure of DNA polymerase *β* due to S44 phosphorylation. Phosphorylation-induced conformational changes transform the enzyme from a closed to an open structure. The dynamic cross-correlation shows that phosphorylation enhances the correlated motions between the different domains. Centrality network analysis reveals that the S44 phosphorylation causes structural rearrangements and modulates the information pathway between the Lyase domain and base pair binding domain. Further analysis of our simulations reveals that a critical hydrogen bond (between S44 and E335) disruption and the formation of three additional salt bridges are potential drivers of these conformational changes. In addition, we found that two of these additional salt bridges form in the presence of Mg ions on the active sites of the enzyme. These results agree with our previous study of DNA pol *β* S44 phosphorylation without Mg ions which predicted the deactivation of DNA pol *β*. However, the phase space of structural transitions induced by S44 phosphorylation is much richer in the presence of Mg ions.

## 1 Introduction

DNA inside the nucleus heavily relies on a complex network of proteins for accurate synthesis and maintenance. Among all the proteins, DNA polymerases are a crucial player. During genome replication and repair, each DNA polymerase catalyzes template-dependent DNA synthesis. DNA polymerase is responsible for preferentially binding and incorporating the nucleotide that correctly base pairs with the appropriate template base. Genomic DNA experiences various damages or modifications during a cell’s lifespan due to physical, chemical, and/or biological insults (Iwanaga et al., 1999; Starcevic et al., 2004; Loeb and Monnat, 2008; Lange et al., 2011). The damage to DNA has wide-ranging consequences for cell function that can result in disease or, in many cases, death. Many aspects of DNA polymerase’s structure-function dynamics of are not fully understood (Johnson, 2010; Mulholland et al., 2012; Walker and Cisneros, 2017).

DNA polymerases are categorized into several families (Arvind and Koonin, 1998; Kunkel, 2003). DNA polymerase (pol) *β* belongs to the X family that includes polymerases involved in different types of DNA repair. Pol *β* is the smallest among all eukaryotic DNA polymerases. It is considered a DNA repair enzyme due to its role in various types of DNA repair, such as Base Excision Repair (BER), and Nucleotide Excision Repair (NER) (Moon et al., 2007).

**FIGURE 1:**
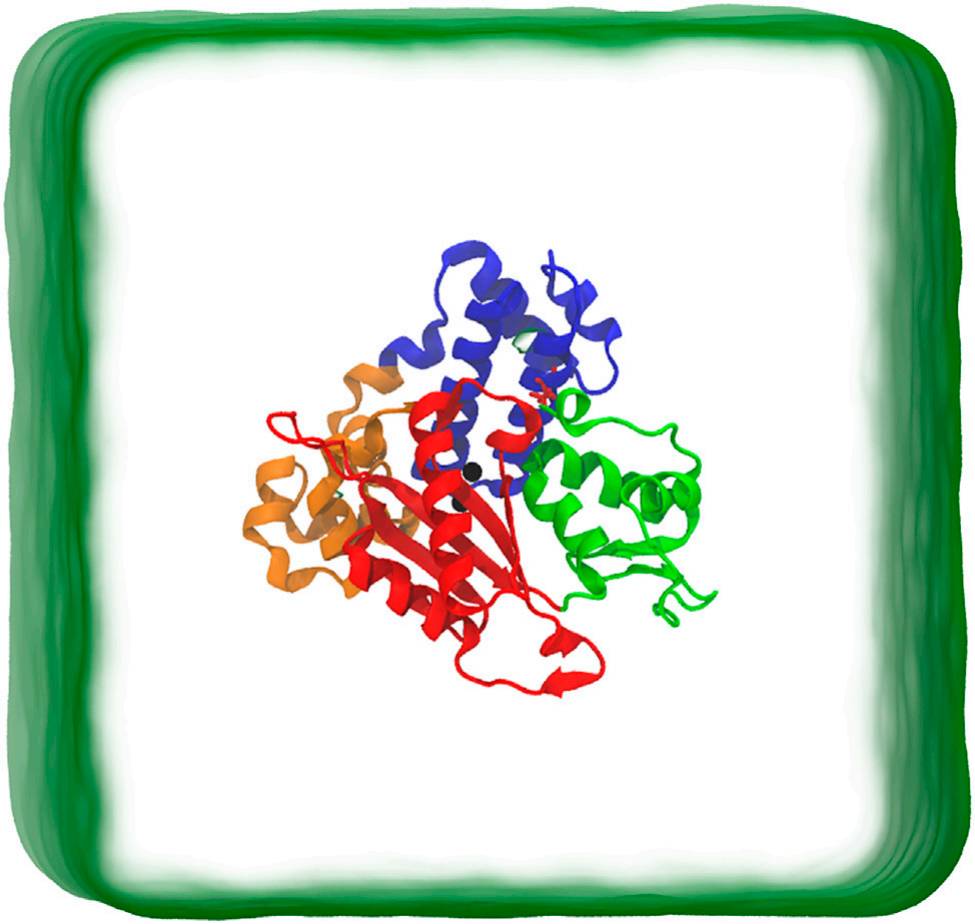
Schematic representation of simulation box. The protein is in closed conformation. The protein structure includes the Lyase domain (residues 10-87) (blue color) and three sub-domains: (DNA binding (D, residues 90-150) (orange color), Catalytic (C, residues 151-260) (red color), and nascent base pair binding (N, residues 261-335) (green color). These correspond to *thumb*, *palm* and *fingers* subdomains, respectively, of DNA polymerases).

The small size and the large amount of available experimental kinetic data (Ahn et al., 1997, 1998; Berg et al., 2001) make DNA pol *β* an attractive model to study the polymerase mechanisms in DNA synthesis. Several high-resolution crystal structures have already been reported for DNA pol *β* and its binary and ternary complexes (Beard and Wilson, 2006; Kohlstaedt et al., 1992; Joyce and Steitz, 1994). DNA pol *β*′s 335 residue has two domains: an 8 kD N-terminal domain that exhibits deoxyribose phosphate lyase activity and a 31 kD C-terminal domain which possesses nucleotidyl transfer activity. The 31 kD domain can be divided into three sub-domains, denoted as *thumb*, *palm*, and *fingers*, as shown in Figure 1 (Joyce and Steitz, 1994; Beard and Wilson, 2006). These three sub-domains correspond to DNA binding (D, residues 90-150), Catalytic (C, residues 151-260), and nascent base pair binding (N, residues 261-335). Various computational methods, especially MD simulations, have been used to investigate the structural changes in DNA pol *β*, such as mutagenic lesions (Florian et al., 2003; Rucker et al., 2009; Meli et al., 2016), and how the polymerase maintains fidelity. These simulations also provide information on how DNA pol *β* transit from the open state to the closed state, but the exact mechanism remains elusive.

MD simulations on kinase protein (Jonniya et al., 2019) show that phosphorylation induced the conformation changes in the protein. Additionally, it is experimentally reported that DNA pol *β* lost its polymerase activity *in vitro* upon serine phosphorylation (S44 and S55) with PKC (Tokui et al., 1991), but not its ability to bind ssDNA. Recently, using MD simulation (Homouz et al., 2018), we showed that the polymerase transit from closed state (active) to open state (inactive) due to S44 phosphorylation. Further analysis reveals that newly formed salt bridges and a hydrogen bond are the probable drivers of these conformational changes. However, the simulations in our previous study did not include Mg ions thought to play an important role in stabilizing the closed state. Two Mg^2+^ ions coordinate with the phosphates of the incoming nucleotide and with Aspartic acid residue (190,192, 256) on pol *β*. Mg ion is necessary for closure of the ternary complex and proper alignment of the incoming dNTP with template nucleotide in the gap that will be filled (Yang et al., 2004). In this work, we investigate the role played by Mg ions and if they can stabilize the phsophorylated enzyme or are distracted from doing so by the newly added phosphate on S44. The role played by Na^+^ ion is not considered since Na^+^ is not a catalytic ion and is not involved in the aforementioned alignment. It coordinates after insertion of correct nucleotide, replacing the catalytic Mg^2+^ while our study is focused on how phosphorylation affects the ternary complex pre-catalysis (Beard, 2020).

Therefore, we build on our earlier study using an improved force field and investigate the role of Mg ions and their possible interference with phosphorylation. Moreover, in this study, we model the phosphorylated serine using two different patches, the dianionic phosphoserine (SP2-) and monoanionic phosphoserine (SP1-) patch. These two patches have one charge difference. The effects of the S44 phosphorylation on conformational changes in pol *β* in the presence of Mg ions are compared between the two patches.

Many MD simulation-based methods (Florian et al., 2003; Arora and Schlick, 2004; Walker and Cisneros, 2017) have been used to study the DNA polymerase fidelity and conformational transitions. Few studies have been devoted to the effect of amino acid mutations on the free energy landscape for proteins (Meli et al., 2016). Moreover, the effect of post-translational modifications on the activity and conformational transitions of DNA pol *β* has not been explored yet. In this work, we performed extensive molecular dynamics simulations to study the conformational transitions of wild-type and phosphorylated DNA pol *β* in the presence of divalent Mg ions. The phosphorylated serine was modeled by using both SP2 and SP1 patches. The main objective of our work is to determine how the phosphorylated serine induces large structural changes in Pol *β* in the presence of Mg ions.

## 2 Materials and Methods

In the present study, the closed form of DNA pol *β* was taken from the crystallographic structure [PDB ID: 2FMS (Batra et al., 2006)]. The DNA was removed from the 2FMS structure to come to a structure that contains only protein. The following systems were investigated: 1)unphosphorylated DNA pol *β*, designated as WT, 2)phosphorylated DNA pol *β*, where SER^44^ was modified to phosphorylated residue from (SP2 patch), designated as pS44, and 3)phosphorylated DNA pol *β*, where SER^44^ was modified to phosphorylated residue from (SP1 patch), designated as p1-S44. The modeled structure included the Mg ions. All the Mg ions were placed at the same positions as resolved in the crystal structure. To understand the mechanism of phosphorylation-induced conformational changes in DNA pol *β*, we have simulated the above systems for 500 ns.

### 2.1 Molecular Dynamics Simulation Setup

All the simulations are performed at constant temperature and pressure (NPT) conditions using the GROMACS 2020 package (Hess et al., 2008). The Charmm36 force field parameters (Best et al., 2012) are used to model protein in addition to the TIP3P water model (Jorgensen et al., 1983). Each system was solvated in a cubic box with a distance of 10 Å from the box walls with TIP3P water molecules. Ions were added to neutralize the system. A time step of 2fs is used for the integration of the equation of motion. Berendsen thermostat was used to set the temperature to 300 K. Pressure was kept constant at 1 bar using the Parrinello-Rahman barostat (Perrinello and Rahman, 1981). Periodic boundary conditions were applied in all directions. The short-range interactions are truncated after 10 Å with a dispersion correction. The neighbor list for non-bonded pairs was updated every 40 steps. A cutoff of radius 10 Å was used for neighbor search. For electrostatics interactions, the Particle Mesh Ewald summation method (Darden et al., 1993) with a grid spacing of 0.16 nm and interpolation of order 4 was used. To constrain the covalent bond length of water and protein to their equilibrium geometries SETTLE (Miyamoto and Kollman, 1992) and LINCS algorithm (Hess et al., 1997) were used respectively. Data is recorded for every 2ps for further analysis. We employed the first steep descent minimization run for 5,000 steps to remove the bad contacts that may come due to the random placement of ions and water. Following minimization, the systems were equilibrated with positional restraint under NVT ensemble at 300 K for 5 ns. Then under constant temperature and pressure, simulations were performed for 5 ns at 1 bar using Berendsen thermostat. Finally, the production run was performed under constant temperature and pressure at 300 K and 1 bar without any restraint. A schematic representation of the simulated system is shown in Figure 1.

### 2.2 Structural Analysis

The built-in function of GROMACS 2020 (Hess et al., 2008) was used to analyze the trajectory produced for each DNA pol *β* system after MD simulation. The results were plotted using the matplotlib v3.3.2 (Hunter, 2007). The structural images were produced using the Visual Molecular dynamics (VMD) program (Humphrey et al., 1996). The gmx rms, gmx gyrate, gmx rmsf, and gmx hbond utilities were used for the structural analysis of each system. We used these utilities to compute the root mean square deviation (RMSD), the radius of gyration (*R*_*g*_), root mean square fluctuations (RMSF), and the number of hydrogen bonds (H-bonds). For the H-bonds, the distance between the donor and acceptor is d ≤3.5 Å, and the angle between the donor and acceptor is $>30^{0}$

### 2.3 Principal Component Analysis

Principal Component Analysis (PCA) (Ichiye and Karplus, 1991) is a popular mathematical tool that is used to analyze the MD trajectory. PCA or Essential Dynamics has been used to reveal the most important information regarding the nature and directions of motion of the proteins.

PCA is based on the diagonalization of the covariance matrix, Cov, with element *Cov*_*ij*_. The covariance matrix built from the cartesian coordinates of the structures obtained from MD simulation:

$$Cov_{ij}=<\left(r_{i}-<r_{i}>\right)\left(r_{j}-<r_{j}>\right)>$$ (1)

where *i* and *j* represent all possible pairs of 3*N* Cartesian coordinates, where *N* is the number of atoms. Here in this work, the PCA was performed on the *C*_*α*_ backbone atoms. The covariance matrix obtained from the atomic fluctuations of protein is diagonalized. The obtained eigenvectors and eigenvalues represent the sets of principal components which could be used to describe the motions. The eigenvalues correspond to the amplitude of motion, whereas the eigenvectors give information about the direction of motions. The first 20 eigenvectors of the system were used to analyze their cosine content in which the two eigenvectors assigned as PC1, and PC2 have a cosine content less than or equal to 0.1. The PC1 and PC2 were used to define the free energy landscape (FEL) (Kumar et al., 2017). The gmx sham built-in function of GROMACS is used to compute the minimum free energy configuration from the PC1 and PC2. The FEL maps were generated using MATLAB.t

### 2.4 Dynamic Cross-Correlation

The cross-correlation coefficient (Hunenberger et al., 1995) is defined as:

$$C\left(i,j\right)=\frac{<\Delta {\text{r}}_{\text{i}}\cdot \Delta {\text{r}}_{\text{j}}>}{{<{\Delta {\text{r}}_{\text{i}}}^{2}>}^{1/2}{<{\Delta {\text{r}}_{\text{j}}}^{2}>}^{1/2}}$$ (2)

where **Δr**_**i**_ is the displacement vector corresponds to atom *i* and the angular brackets denote an ensemble average. Details are summarized in supplementary information (SI).

The positive value of *C*(*i*, *j*) inferred correlated motions, whereby the two *C*_*α*_ atoms moved in the same direction, whereas the negative value implies anti-correlated motions, whereby the two atoms moved in the opposite direction. *C*(*i*, *j*)= 1, means the motions is completely correlated or *C*(*i*, *j*)= −1, the motion is completely anti-correlated. The elements of the cross-correlation metric do not have any information about the magnitude of the motion of the atoms. Complete correlation means the two atoms are moving with the same phase and period. If two atoms move with the same period and phase but their displacements are oriented at an angle of 90°, then *C*(*i*, *j*) = 0.

### 2.5 Structural Network Analysis

In our residue interaction network, each node represents the *C*_*α*_ atom of each residue. Dynamic cross-correlation matrix elements can be used to compute the weight. The weight corresponds to the probability of information transfer across the edge. Each edge in our network has an information transfer probability. The weight of the edge between the node *i* and *j* is defined as (Sethi et al., 2009):

$$\omega_{ij}=-\log|C_{ij}|$$ (3)

To identify the critical residues responsible for information flow, we computed the three different centrality measures, degree, betweenness, and closeness centralities, from the residue interaction network (Freeman, 1978; Newman, 2005; Mason and Verwoerd, 2007). The degree centrality (*C*_*d*_) calculated is the number of edges connected to a node in a network.

$$C_{d}\left(r_{i}\right)=d_{i}=\underset{j}{\sum }M_{ij}$$ (4)

where *M*_*ij*_ is the matrix: if *ω*_*ij*_ = 0 then *M*_*ij*_ = 0, otherwise *M*_*ij*_ = 1.

The closeness centrality (*C*_*c*_) calculates the average distance of the shortest paths between a node and all the other nodes in a network. Thus, it computes the information flows from a given node to the other nodes. The closeness centrality is defined as

$$C_{c}=\frac{N-1}{\sum_{j\neq i}d\left(i,j\right)}$$ (5)

where N is the chain length and d is the shortest path with a weight between two nodes, *i* and *j*.

The betweenness centrality calculates the total number of information pathways that flow through a node in a network. The betweenness of node *i* is the fraction of the shortest paths between pairs of nodes that pass through node *i*. The betweenness centrality is defined as

$$C_{B}=\frac{2}{N\left(N-1\right)}\underset{a<b}{\sum }\frac{\tau_{i}^{ab}}{N_{ab}}$$ (6)

where $\tau_{i}^{ab}$ is the number of shortest paths from *a* to *b* with a weight that passes through node *i*. *N*_*ab*_ is the total number of shortest paths from *a* to *b*. Details are presented in SI. All the three centralities are computed using the Bio3d package (Grant et al., 2021).

## 3 Results and Discussion

DNA polymerase *β* visits several intermediate states along its reaction pathway (Walker and Cisneros, 2017). The unliganded enzyme has an extended structure [PDBID:1BPD (Sawaya et al., 1994)]. It adopts an open structure after binding DNA [PDBID:3ISB (Beard et al., 2005)], which undergoes further conformational changes to form the closed structure after binding dNTP [PDBID:2FMS (Batra et al., 2006)]. In this work, we studied the effect of phosphorylation on the conformational transition of the closed state of DNA pol *β* using extensive MD simulation in the presence of Mg ions. The phosphorylated related biochemical study has been performed on rat DNA pol *β*, but in this study, we used the human DNA pol *β* for MD simulation.

### 3.1 Structural Stability and Flexibility Analysis

**FIGURE 2:**
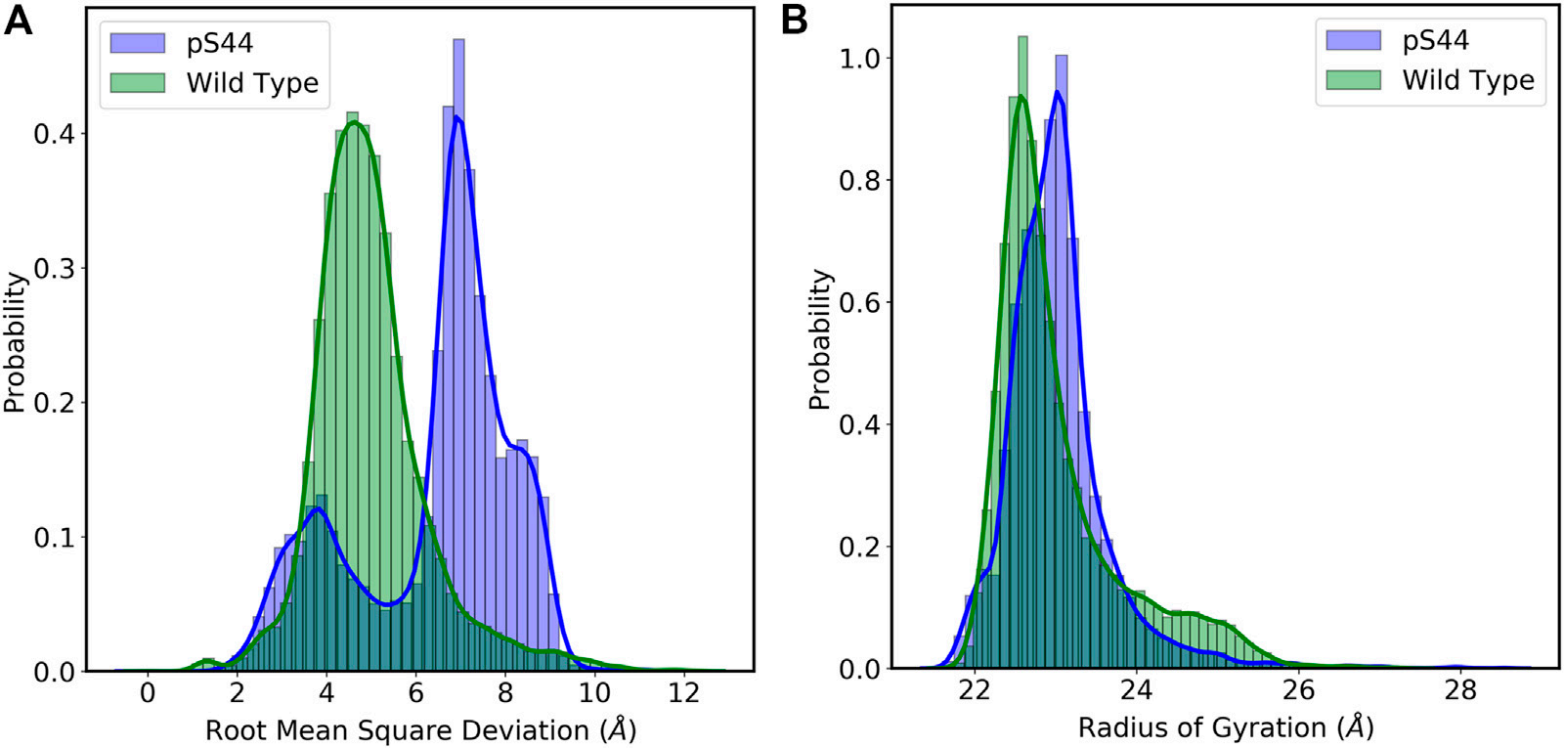
Probability distribution of **(A)** Root mean square deviation (RMSD): Wild type (green), and pS44 (blue) **(B)** Radius of gyration (*R*_*g*_): Wild type (green), and pS44 (blue).

To assess the structural changes in the enzyme, we computed the RMSD of the *C*_*α*_ atoms from the initial structure. RMSD calculations will also enable us to evaluate the overall stability and convergence of simulations. Figure 2A shows the frequency distribution of RMSD of WT and pS44. The figure shows that the distribution for the WT system has one peak (∼ 4.5 Å), whereas that of the pS44 system has two peaks (∼ 3.7 Å and 7.14 Å). The average value of RMSD for WT is 4.98 Å, whereas, for pS44, the average RMSD is 6.42 Å. This clearly shows that the enzyme becomes more flexible upon phosphorylation.

The phosphorylation effect on the structural compactness of the enzyme can be examined by computing the *R*_*g*_. The frequency distribution of *R*_*g*_ for both cases is shown in Figure 2B. The frequency distribution of *R*_*g*_ shows a single peak in both cases; this means the domains are tilted or twisted away from their equilibrium position but don’t completely open. The average value of *R*_*g*_ for WT is 23.04 Å, whereas, in the case of pS44, a slightly higher value of 23.15 Å was obtained. To further analyze the peaks in Figure 2, we calculate the free energy landscape (FEL).

**FIGURE 3:**
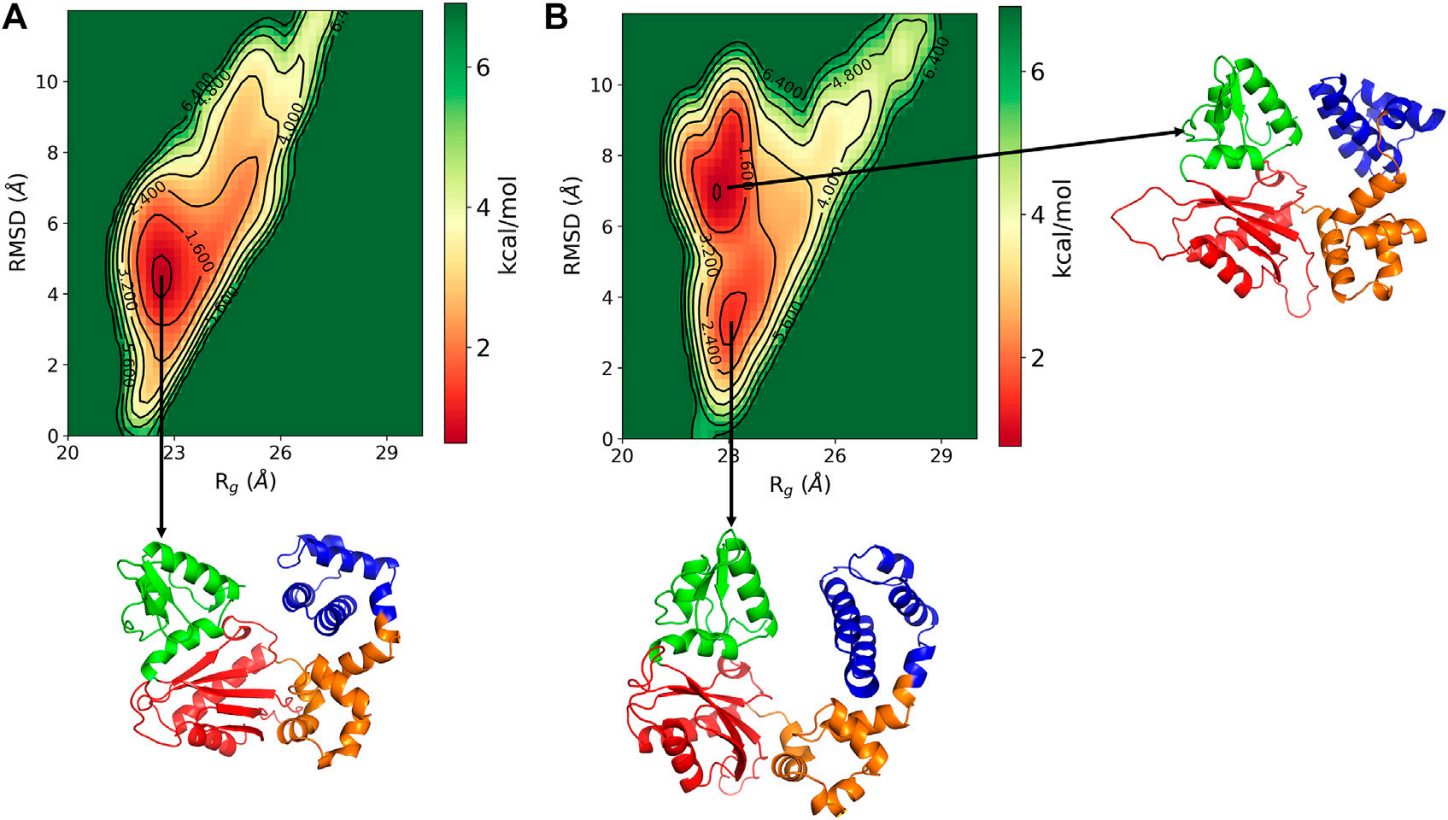
Free energy landscape of DNA polymrase *β* as a function of *R*_*g*_ (Å) and RMSD (Å) for **(A)** WT and **(B)** pS44. Representative structure corresponds to the minimum energy states are also shown.

The conformational changes in the DNA pol *β* are explored by the free energy landscape (FEL). The FEL is plotted in terms of RMSD and *R*_*g*_ of the enzyme, which is shown in Figure 3. The FEL describes the conformational space explored by the enzyme. It shows that in pS44, there are two major minimum free energy basins that characterized by rmsd ∼3.56 Å/*R*_*g*_ ∼23.11 Å and rmsd ∼7.1 Å/*R*_*g*_ ∼22.7 Å, whereas for WT, there is a single broad minimum free energy state explored with rmsd ∼4.54 Å/*R*_*g*_ ∼ 22.6 Å, implying that more deviation for pS44 compared to WT. The snapshots corresponding to the free-energy basins for both cases were shown in Figure 3A WT; and Figure 3B pS44 (Supplementary Figure S1 (in SI) for WT in absence of Mg ions). It is clear from the figure that due to the phosphorylation, the system visits the two conformational states corresponding to the two peaks in Figure 2A. These two states differ from each other by changes in the Lyase domain of the enzyme. The Lyase domain swing towards the C sub-domain in the first basin. On the other hand, the Lyase domain is slightly twisted and slides down the N sub-domain away from E335 in the second basin compared to the closed state of the enzyme. These changes indicate that the Lyase domain is functionally and structurally affected by phosphorylation; however, this was not examined experimentally *in vitro*. The conformational changes induced by phosphorylation don’t have a big effect on the value of *R*_*g*_ since they involve movements of the Lyase domain towards other sub-domains.

**FIGURE 4:**
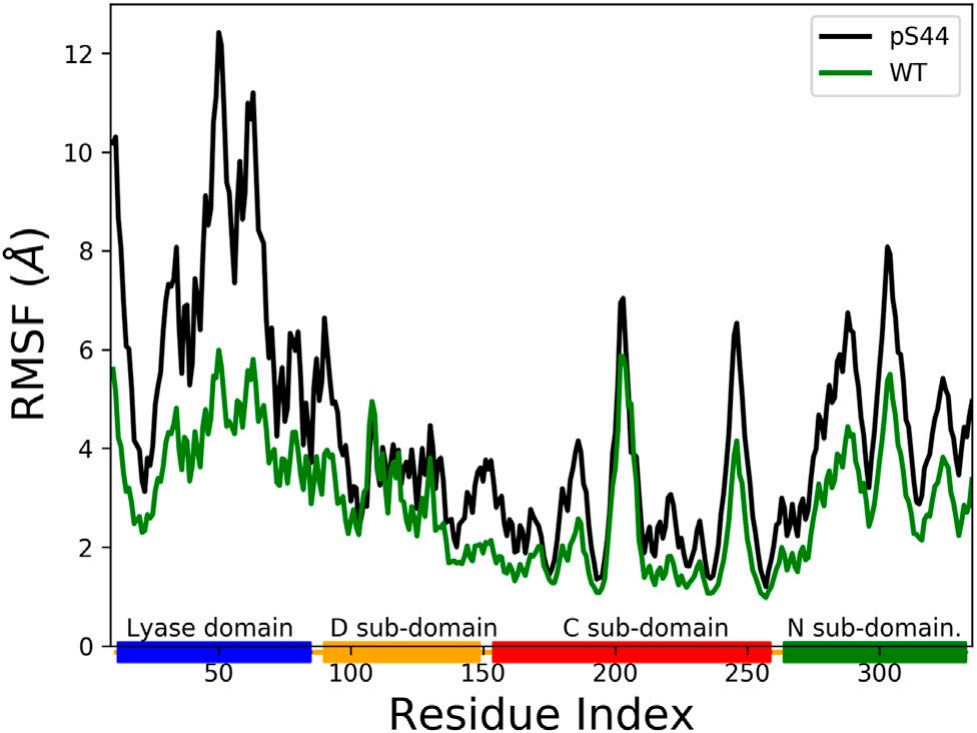
The Root Mean Squared Fluctuation (RMSF) for pS44 (black), and WT (green). The rectangle’s shown in horizontal panel reflects the DNA polymerase *β* major sub-domains, are colored as follows: Lyase domain (blue) and three sub domains: (D (orange), C (red), and N (green).

The phosphorylation at S44 induces large conformational changes in the closed structure. To find the domains affected by the phosphorylation, we computed the root mean square fluctuation (RMSF) for *C*_*α*_ atoms of protein residues. The RMSF value shows the average fluctuation of each residue over the total time of the simulation. Figure 4 compares the RMSF of pS44 with that of WT, which shows that phosphorylation (S44) induces large structural fluctuations in the Lyase domain and the N sub-domain.

The DNA polymerase experimentally resolved crystal structure indicate that the enzyme goes into a closed state due to the presence of a hydrogen bond (H-bond) between S44 and E335 residues (Batra et al., 2006). This H-bond is missing in an open state. This indicates that this interaction is responsible for stabilizing the enzyme in the closed state. To analyze the effect of phosphorylation of S44 on this bond, we calculated the H-bond occupancy in the MD simulation. The H-bond occupancy is defined as the fraction of time in which the S44 residue forms the H-bond with the E335 residue. The H-bond was calculated using the gromacs hbond tool that determines the presence of the H-bond based on a cutoff distance of 3.5 Å and a cutoff angle of 30°. The result shows that phosphorylated S44 disrupts the H-bond, and occupancy remains 0.0 throughout the trajectory. Supplementary Table S1 (in SI) shows the average donor-acceptor distance between S44 and E335 for all simulated systems. The H-bond disruption might be associated with the negative charge of the phosphate group. To study the effect of phosphorylation of S44 on polar interactions, we have computed the H-bonds formed in between the pS44 residues and the different domains of DNA pol *β*, and we found that they varied throughout the simulation. Notably, the maximum number of H-bonds between the Lyase and pS44 residue is 9; pS44 residue and D sub-domain is 3; pS44 residue and N sub-domain is 2. The C sub-domain does not form any H-bonds with the pS44 residue. Since the pS44 residue exists in the Lyase domain, therefore it forms a large no of H-bonds with the neighboring residues. Supplementary Figure S2A (in SI) shows the time evolution of Hydrogen bond forms between the Lyase domain and Serine 44 for WT and pS44 whereas Supplementary Figure S2B shows the time evolution of hydrogen form between the D sub-domain and S44 for pS44. It is clear from the figure that the pS44 residue forms more H-bonds compared to the S44 residue in WT, consistent with the literature (Mandell et al., 2007; He et al., 2016). Thus, H-bond analysis signifies the role of hydrogen bonds in opening up the structure of the pS44 system.

### 3.2 Salt Bridges Formed and the Role of Mg Ions

In addition to disrupting the hydrogen bond between the S44 and E335, phosphorylated S44 is expected to form new salt bridges with the surrounding residues. We performed the salt bridge analysis for pS44. The phosphorylated S44 forms four salt bridges (with R40, K41, K48, and R149) in the absence of Mg ions. However, six salt bridges form with residues (R40, K41, K48, R149, K280, and R299) in the presence of Mg ions. The first four salt bridges (with R40, K41, K48, and R149) are shown in Supplementary Figure S3 (in SI). The first three residues (R40, K41, and K48) lie in the Lyase domain and the salt bridge formation with them is not likely to cause major conformational changes due to the fact the O-N distances between S44 and these residues are not large in the WT system in the absence or presence of Mg ions. For example, in the WT system in the presence of Mg ions, the average distance between the S44 and R40 is 7.52 Å; S44 and K41 is 7.27 Å; and S44 and K48 is 6.86 Å, whereas for pS44 system, the distance between the pS44 residue and the residues are 4.46 Å, 5.89 Å, and 3.62 Å. The other three salt bridges (with R149, K280, and R299) which form post phosphorylation in the presence of Mg ions are more significant since the corresponding residues are located far from S44 in the WT system. The R149 lies in the coil region, which connects the D sub-domain to the C sub-domain. The formation of the salt bridge with R149 is consistent with the predictions of our previous study (Homouz et al., 2018).

**FIGURE 5:**
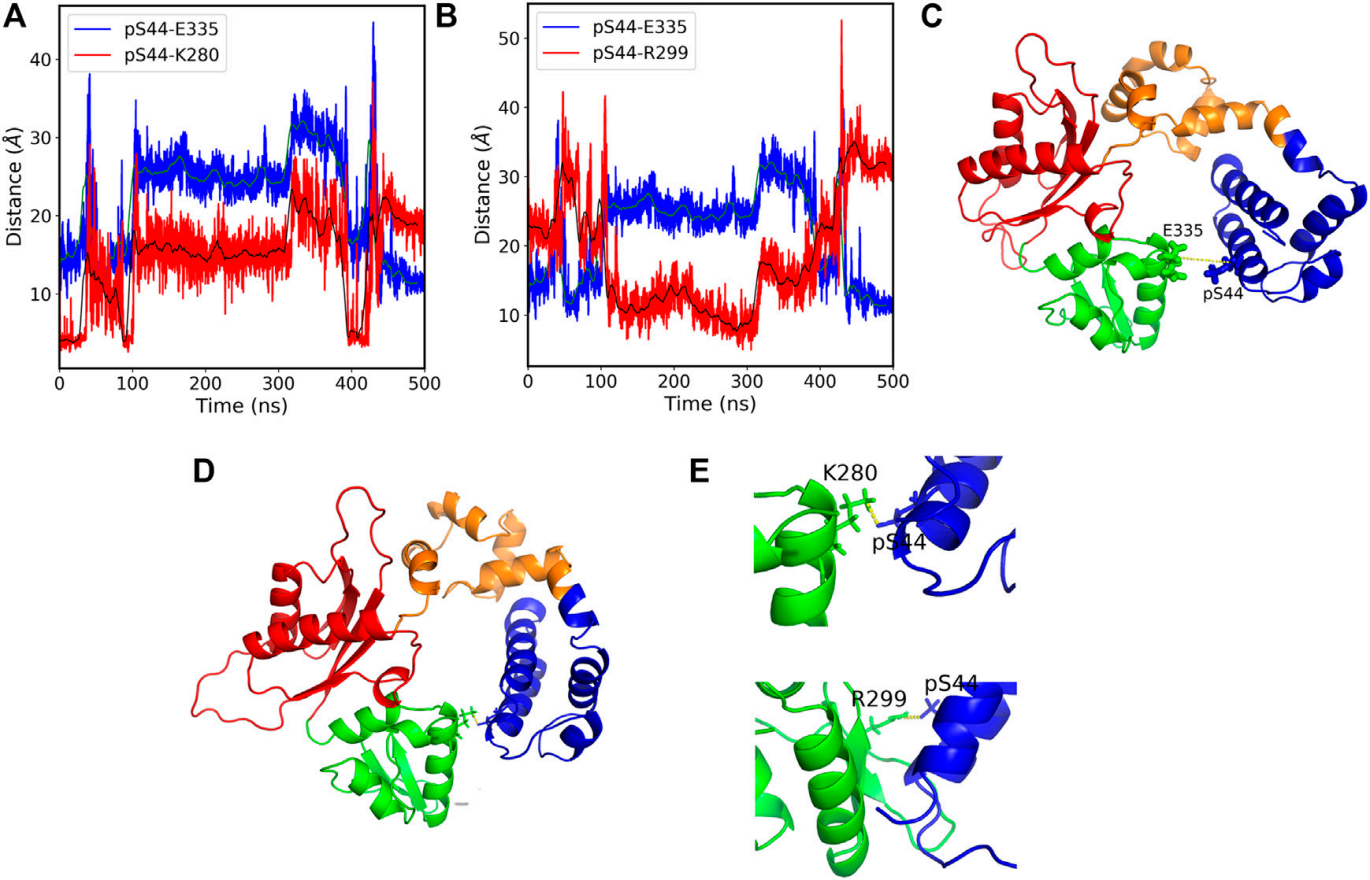
**(A)** The pS44-E335 (blue) and pS44-K280 (red) residue distances *vs*. simulation time. The figure shows that the distances between the residues correlate with each other over a large section of the simulation time. the green and black solid lines show the moving average of data with a bin size of 500. **(B)** The pS44-E335 (blue) and pS44-R299 (red) residue distances *vs*. simulation time. The figure shows that the distances between the residues anti-correlate with each other over a large section of the time. **(C)** Cartoon representation of pS44-2FMS structure. The pS44 and E335 residues are shown in stick representation. **(D)** Cartoon representation of salt-bridges formed with K280 and R299. The residue pS44, E335, K280, and R299 are shown in stick representation. **(E)** Salt bridges formed with K280 and R299 are shown by zooming the structure. The figure shows that Lyase domain twist and pS44 come close to R299 and form a salt bridge. DNA polymerase *β* major sub-domains are colored as mentioned in Figure 1.

The salt bridges with the residues (K280 and R299) which lie in the N sub-domain form only in the presence of Mg ions. Hence, our results suggest that phosphorylation leads to the formation of new salt bridges that leads to conformational changes. Moreover, considering the two additional salt bridges formed due to the phosphorylation in the presence of Mg ions, we see that the pS44-K280 (O-N) distance correlates with the pS44-E335 distance (Figure 5A), whereas the pS44-R299 salt-bridge anti-correlates with pS44-E335 distance (Figure 5B). This anti-correlation is due to the structural rearrangements of the Lyase domain and C sub-domain (Figures 5C,D). The salt bridges between pS44-K280 and pS44-R299 are mapped on the structure shown in Figure 5E. Thus, the formation of these new salt bridges (K280 and R299) is another driving force behind the major conformational changes induced by pS44. We suspect that the presence of Mg ions plays a major role in facilitating these salt bridges. To elucidate this role, we analyzed the interactions of Mg ions at the active sites (D190, D192, and D256) with the surrounding residues. In the absence of Mg, the residues at these active sites form a network of salt bridges with several other residues (R149, K234, R254, and R258) that make the polymerase domain more rigid (see Supplementary Table S2 and Supplementary Figures S4, S5). In the presence of Mg ions, the salt bridges with (R149 and K234) are lost probably due to the screening effect of the positive charge on Mg ions. The loss of these salt bridges makes the polymerase domain more flexible in the coil region which could allow for the N sub-domain to hug the lyase domain. Such structural flexibility might well explain the formation of the new salt bridges between pS44 and the two residues in the N sub-domain. Molecular dynamics suggested that phosphorylation may influence the overall hydrophobicity for a protein (Polyansky and Zagrovic, 2012) and that loss of folding due to altered hydrophobicity may be compensated for by salt-bridges (Seshasayee et al., 2005). It is possible that the salt bridges in our models serve this purpose.

### 3.3 Correlated and Anti-Correlated Motions

**FIGURE 6:**
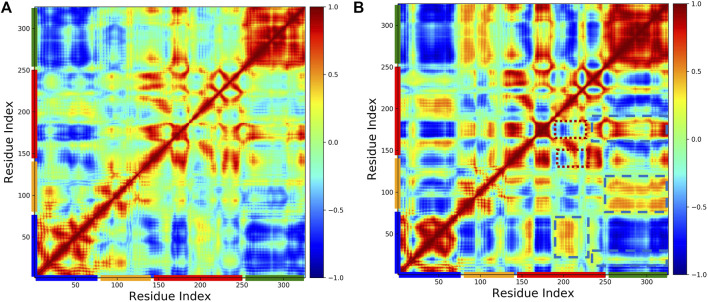
Cross-correlation matrices of the fluctuation of C-alpha atom around their equilibrium positions for WT and phosphorylated WT are shown in **(A)** WT, and **(B)** pS44, respectively, from simulations. The correlated and anticorrealted motions are color coded (highly correlated =1, no correlated =0, anticorrlated =-1).

To investigate the effect of phosphorylation on the domain motions of pol *β*, we generated the dynamic cross-correlation maps using the method described above. Figure 6 shows the cross-correlation map (A) WT and (B) pS44. It is clear from the figure that S44 phosphorylation increases the correlated motions between the different domains. For the pS44 system, most residues between different domains are highly correlated except for a few residues in the C sub-domain (highlighted in red dotted line square). In the case of WT, all the intra-domain motions are highly correlated. Looking at the overall correlation motions between pairs of domains, we see that the Lyase-N sub-domain motion is not correlated in both systems, whereas the Lyase-D sub-domain is more correlated in WT compared to pS44. On the contrary, the Lyase-N sub-domain is correlated in the pS44 system and not correlated in the WT system. In pS44, the inter-domain correlated motions are observed more in different regions (highlighted by blue dashed line square) compared to WT. When the S44 is phosphorylated, the anti-correlated motions are seen in the N sub-domain and N sub-domain (highlighted by a red dotted line square), which is not observed in WT. Overall, correlated as well as anti-correlated domain motions were modified after the phosphorylation of S44. In particular, all the interactions explained in our previous section were justified with the cross-correlation map, such as the salt bridges.

### 3.4 Principal Component Analysis

We performed the principal component analysis (PCA) by diagonalizing the covariance matrix of atomic fluctuation for WT and pS44. We obtained the results in terms of eigenvalues (3N, 3 × 326 =978) versus eigenvectors. The first 20 eigenvalues both for WT and pS44 are shown in Supplementary Figure S6A, B (see SI). The eigenvalues were plotted in decreasing order. The first few eigenvalues correspond to the largest fluctuations of the protein. Comparing the two systems, we find that the first few principal components that describe the cooperative behavior of motion were not the same, and in pS44, the magnitude of the first few eigenvalues was higher compared to WT. The first 20 eigenvectors describe the collective motion of protein and approximately account for ∼ 98, and 95% overall motions in pS44 and WT, respectively. The first two eigenvectors capture around ∼ 79 and ∼ 55% of total motions in pS44 and WT, respectively, even for pS44, the first principal component is enough to describe 50% of the overall motion of pS44. Our results suggest that the correlated motions were higher for pS44 in agreement with the cross-correlation results.

**FIGURE 7:**
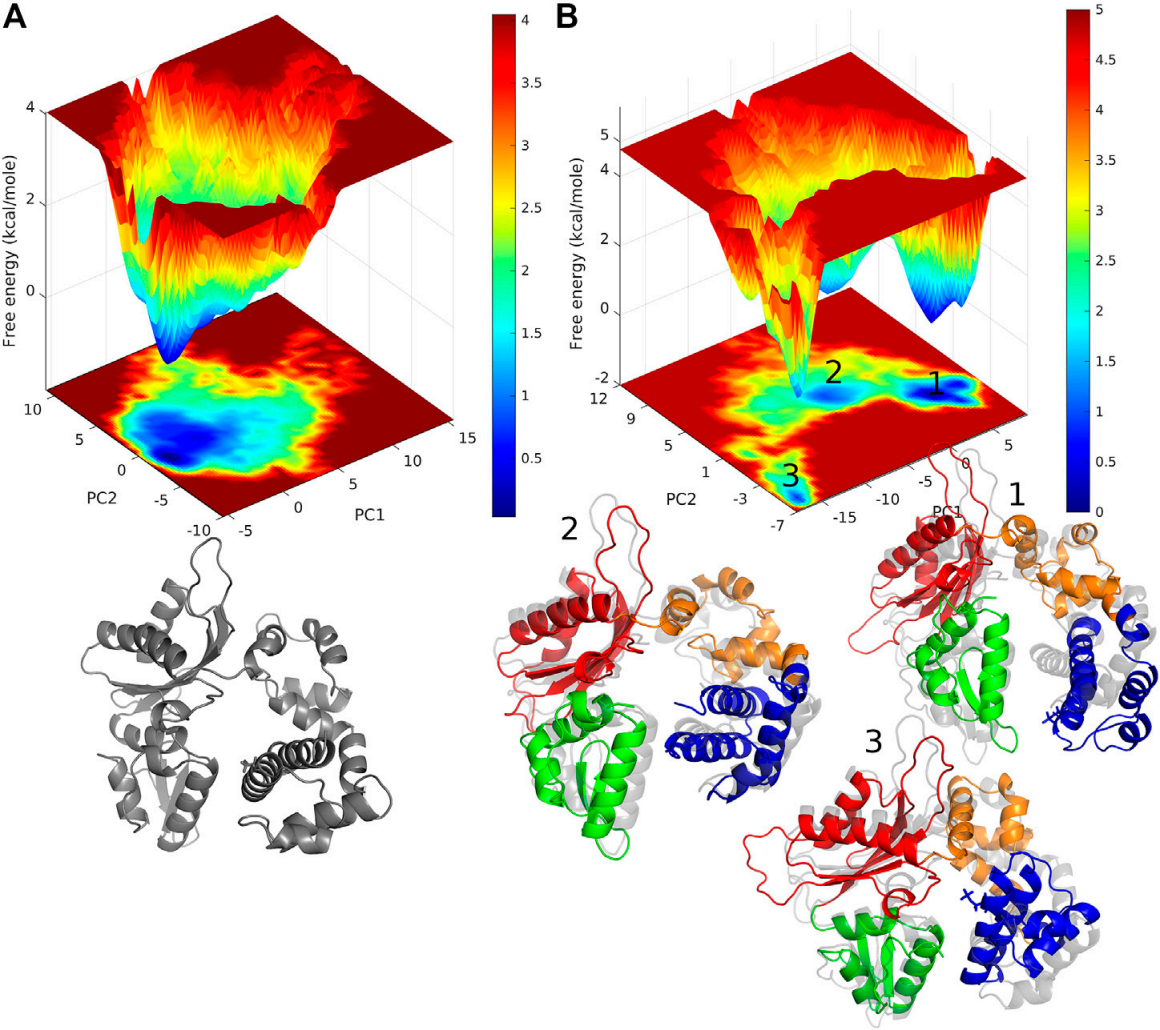
free energy landscape generated by projecting the PC1, and PC2 of **(A)** WT, and **(B)** pS44, whose cosine content was less than 0.1. Representative structure corresponds to the minimum energy states are also shown. For pS44, the minimum energy basins are highlighted using the numbers.

Free energy landscape (FEL) analysis will give us information about the minimum energy conformations. The FEL contour maps for WT and pS44 were derived from the first two principal components (as mentioned in the methodology). FELs for both systems are represented in Figure 7. The patterns of the free energy basins on the FELs are different between the two systems, as apparent from Figure 7. In the case of pS44, the conformational space shows multiple minimum free energy basins and large structural distributions, whereas, in the case of WT, there is only a single broad and global stable minimum observed on the free energy surface. The three basins of the pS44 free energy landscape are labeled 1, 2, and 3. The figure shows snapshots of the structures in these three basins contrasted with the WT basin. As we can see from these snapshots, basin 1 indicates a pS44 system with the Lyase domain twisted at the linker away from the N sub-domain. Basin 2 corresponds to a structure that opens up around the Lyase and N domains, while in basin 3, the Lyase domains swing out such that the S44P residue moves closer to the D sub-domain. The results suggest that the enzyme attains more conformational flexibility and dynamics with phosphorylation.

**FIGURE 8:**
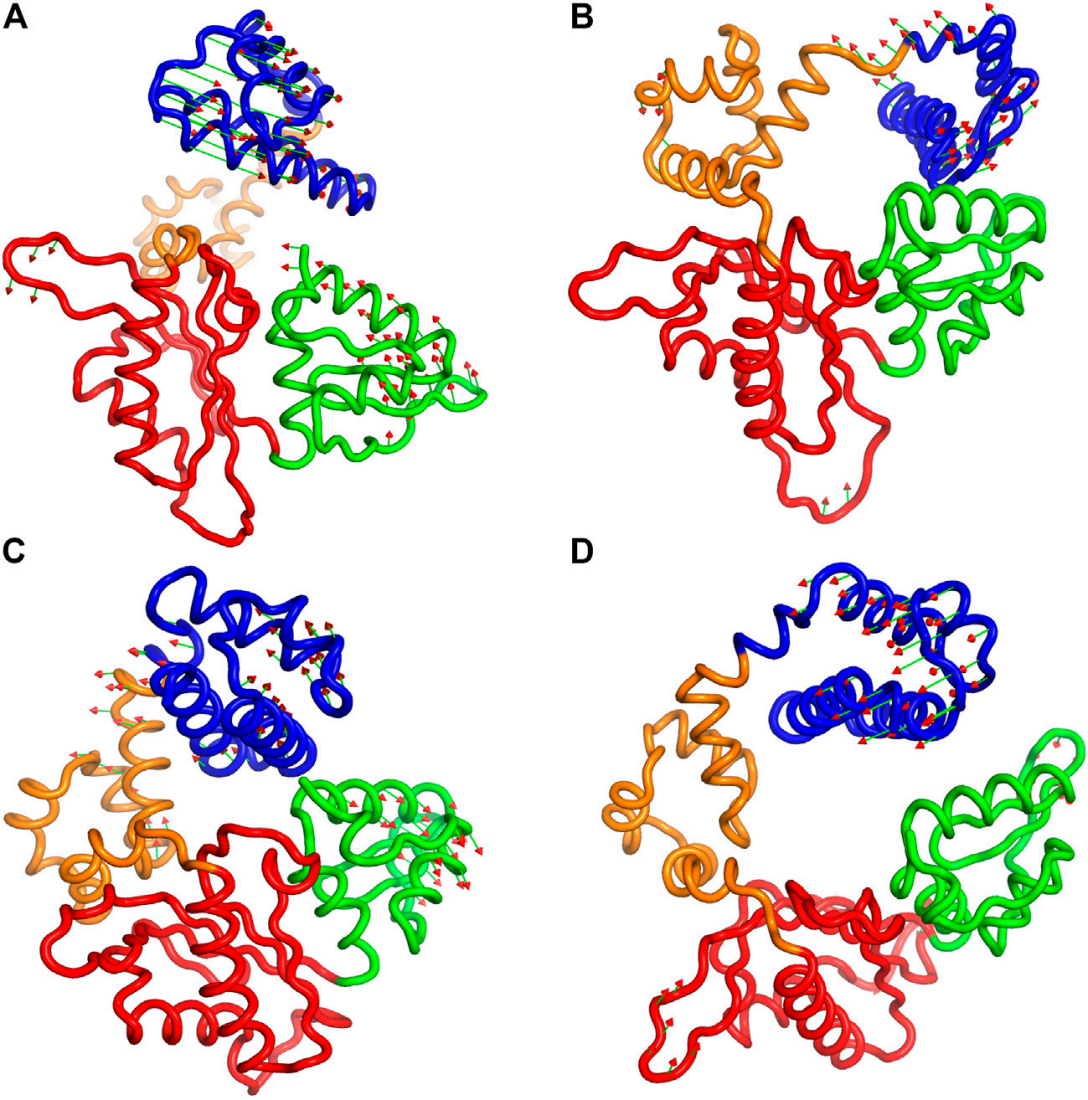
Procupine plots showing prominent motion for **(A)** pS44 (PC1), **(B)** pS44(PC2), **(C)** WT (PC1), and **(D)** WT (PC2). The arrow represents the eigen-vectors showing the direction of prominent motions of pS44 and WT, respectively.

Furthermore, to illustrate the effect of phosphorylation on the direction and magnitude of motions, the eigenvectors corresponding to mode 1 and mode 2 were visualized by using “Porcupine plots” for both pS44 and WT. Porcupine plots were generated using Pymol (Schrödinger and DeLano, 2015) shown in Figure 8. The plots correspond to the first and second modes, which offer the largest collective motions of *α*-carbon atoms had been mapped onto the average structure. The cutoff of mode vectors was chosen 15 Å for easy visualization. Figure 8 shows that the overall direction of motions of the lyase domain and N sub-domain changes after phosphorylation which agrees with the FEL plots (Figure 7) and RMSF plot (Figure 4). The figure shows that the first principal component, PC1 and PC2, of pS44 correspond to twist and opening of the Lyase domain, respectively. Furthermore, Supplementary Figure S7 (in SI) shows the residue-wise contribution of PC1, and PC2 for both p-WT and WT. Overall, it is observed that the mobility is higher for pS44 compared to WT, as can be ascertained.

### 3.5 Network Centrality Analysis

Computing different centralities is a popular concept in social network analysis, which could increase the relevance of selecting critical nodes in the network. Centrality analysis has been successfully applied in proteins to identify the functionally important residues in allosteric communication, metabolic network, and disease network (Amitai et al., 2004; Whitley and Lee, 2009; Ruvo et al., 2012). The three most widely applied centrality measures are degree (*C*_*d*_), closeness (*C*_*c*_), and betweenness (*C*_*B*_). To identify the critical residue responsible for information flow and how the phosphorylation affects the information flow, we constructed the residue interaction network and calculated the different centrality measures using the last 300 ns of the trajectory. To show the effect of phosphorylation, we computed the centralities for both WT and pS44 systems. To check the correlation between the different centralities, we computed the Pearson correlation coefficient between the three different centralities, which lies in the range of 0.6–0.72. Hence a residue with a high *D* or high *C*-value does not necessarily possess a high *C*_*B*_-value.

**FIGURE 9:**
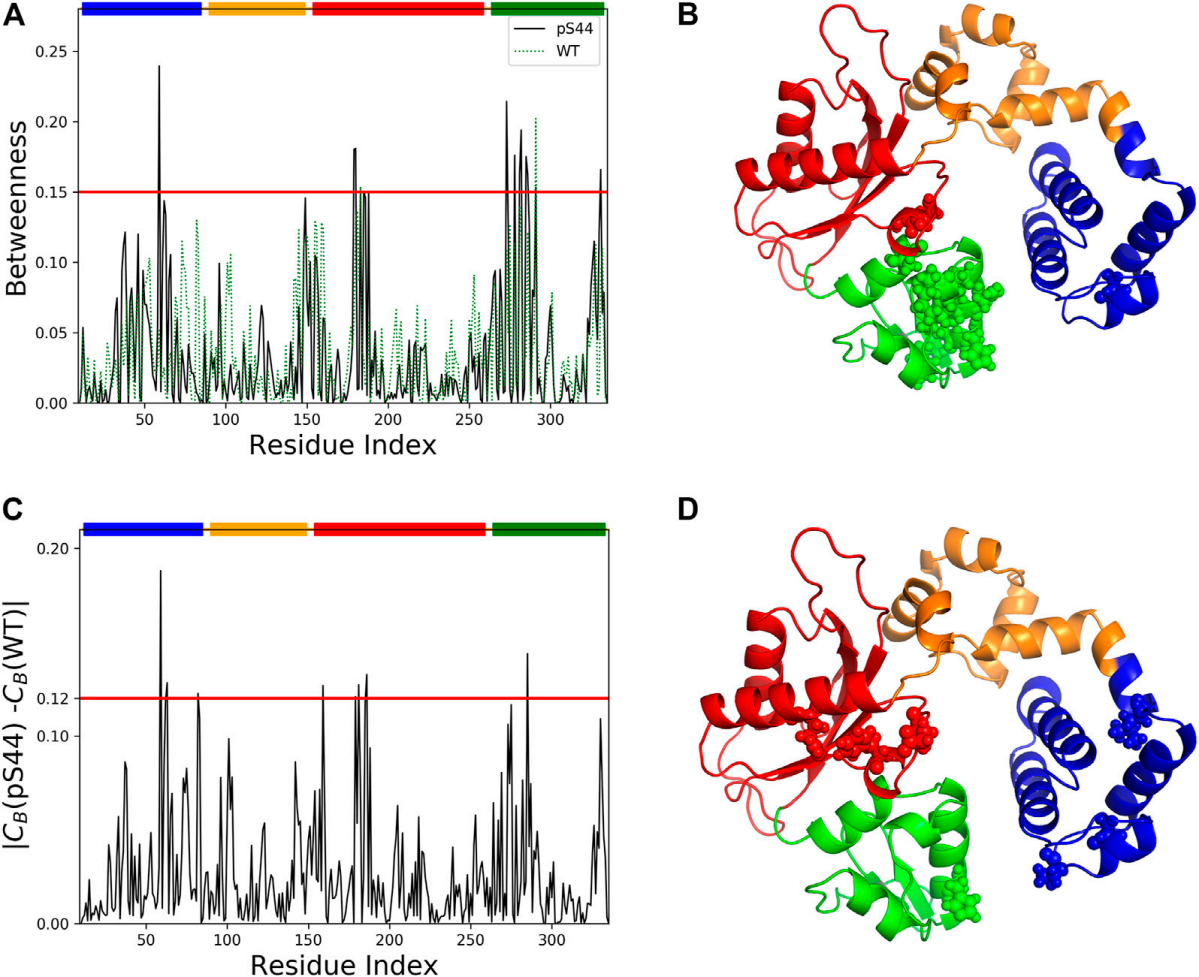
Network centrality analysis for WT and pS44. **(A)** Betweenness centrality for each residue of pS44 and WT are shown. **(B)** Residues with high betweenness value (≥0.15) are shown in sphere representation on the minimized pS44 structure. Difference of betweenness value calculated for **(C)** pS44 and WT. **(D)** Residues with|*C*_*B*_(*pS*44) − *C*_*B*_(*WT*)| ≥ 0.12 depicted on minimized pS44 structure as spheres representation. The colors of the sphere are according to DNA pol *β* domain, mentioned in Figure 1.

Previous studies (Lee et al., 2014) have shown that a residue with a high *C*_*B*_-value correlates well with the functional residue that mediates the allosteric signal. This indicates the importance of *C*_*B*_ over two other centrality measures. Hence, we used *C*_*B*_ to measure the relevance of each residue for the signal flow in different systems. Supplementary Figure S8 (in the SI) shows *C*_*d*_ and *C*_*c*_-values per residue for WT and pS44. The results show that after phosphorylation, both centrality measures increase. Figure 9 shows the betweenness value per residue for WT and pS44. It is clear from the results that the *C*_*B*_-value in the Lyase domain and N sub-domain increases after the phosphorylation, which is consistent with RMSF-value and correlation map.

To visualize the information flow in enzyme structure, we mapped the residue with *C*_*B*_-values ≥ 0.15 onto the minimized for of phosphorylated DNA pol *β*. A total of 14 residues were identified using *C*_*B*_ ≥ 0.15. These residues are distributed contiguously in the Lyase and N sub-domain and thus could be assumed crucial for modulating the signal flow.

We also computed the differences in the *C*_*B*_ between the WT and pS44 forms (Figure 9). The residues satisfying the condition |*C*_*B*_(*WT*) − *C*_*B*_(*pS*44)| ≥ 0.12 are mainly located in the Lyase and N sub-domain, also satisfying *C*_*B*_ ≥ 0.15. For visualization purposes, the residues that satisfy the condition |*C*_*B*_(*WT*) − *C*_*B*_(*pS*44)| are shown in the minimized enzyme structure.

### 3.6 DNA Pol Beta Modeled With SP1 Patch Transforms Into Extended State

The serine phosphorlyated *in vitro* by Tokui et al. (1991) can be modeled accurately by the SP2 patch in our simulations. Therefore, our main emphasis is on conformation transitions observed in S44 phosphorylated DNA pol *β* modeled by the SP2 patch. However, using a different patch, namely SP1, could help us gain more insight into the role that charges play. Since the two phosphorylated serine patches differ in the charge, therefore, we compare the different conformational states visited by pS44 corresponding to the two different patches. The conformational changes in the DNA pol *β* are described by the free energy landscape (FEL) in terms of RMSD and *R*_*g*_ of the enzyme is shown in Supplementary Figure S9 (see in SI). The FEL shows that due to the phosphorylation, the DNA pol *β* goes into an extended state. We observed the two free energy basins in out FEL characterized by rmsd ∼3.7 Å/*R*_*g*_ ∼23.3 Å and rmsd ∼17.2 Å/*R*_*g*_ ∼31.6 Å. In the SP2 patch, the DNA pol *β* goes into an open state due to phosphorylation, whereas using the SP1 patch, the enzyme adopts an extended state. The snapshots corresponding to the free energy basins are shown in Supplementary Figure S9. As we know from our SP2 patch analysis, newly formed salt bridges play a major role in the conformational transition observed in DNA pol *β*. The newly formed two additional salt bridges help the system to transit from closed to open state. The results show that the SP1-phosphorylated S44 forms salt bridges with (R40, K41, K48). The two key salt bridges with R149 and R299 that are present in the SP2-pS44 system and help stabilize the open state are missing in the SP1-pS44 system. Moreover, the salt bridge with K280 is weakened significantly with SP1-phosphorylated S44. The loss of these key salt bridges destabilizes the closed structure and facilitates its transition into the extended state.

## 4 Conclusion

In this work, MD simulations were performed to investigate the effect of Ser44 phosphorylation on the structural dynamics of the DNA pol *β*. Our previous preliminary results (Homouz et al., 2018) suggested how Ser44 phosphorylation may cause deactivation of polymerase function for DNA pol *β*. The simulations reported here are designed to further analyze the probable mechanism behind this deactivation by using longer simulations times, improved force-field, and novel structural analysis methods. Furthermore, the simulations investigate the role played by the coordinating Mg ions and their possible effects on phosphorylation-induced changes. Simulation results suggest that S44 phosphorylation leads to conformational transitions in the enzyme and affects the motions of different segments of the enzyme such as the Lyase domain, and base pair binding sub-domain. Additional stable salt bridges are observed upon phosphorylation. The key Hydrogen bond between S44-E335 was disrupted after phosphorylation. Network centrality analysis reveals that phosphorylation causes structural rearrangements and modulates the communication path between the Lyase domain and N sub-domain. Free energy landscapes of the enzyme visit multiple stable conformational basins for the phosphorylated variant.

The simulations show that in pS44, the lyase domain twists and moves closer to N sub-domain and forms salt bridges with residue K280 and R299 and drives the system into an open state. The Mg ions play a major role in the formation of these two new salt bridges which were missing in our earlier study. Using the SP1 patch, we showed that the enzyme goes into the extended state due to the disruption of the S44-R149 salt bridge. Our results confirm that phosphorylated serine 44 leads to significant conformational changes in DNA pol *β* that affect its activity.

## Data Availability

The raw data supporting the conclusion of this article will be made available by the authors, without undue reservation.
