# Supplementary material for: Phosphorylation Induced Conformational Transitions in DNA Polymerase β
Source: Front Mol Biosci. 2022 Jun 13;9:900771. doi: 10.3389/fmolb.2022.900771 (PMC9234555; doi:10.3389/fmolb.2022.900771)
Supplement: Supplementary file 1 [file DataSheet1.PDF]

Supplementary Material

1 DYNAMIC CROSS-CORRELATION

The cross-correlation coefficient between the ith and j^{th} atoms is defined by the following equation,

$$C(i,j) = \frac{< (\mathbf{r_i(t)} - < \mathbf{r_i(t)} >) \cdot (\mathbf{r_j(t)} - < \mathbf{r_j(t)} >) >_t}{(< \mathbf{r_i^2(t)} > - < \mathbf{r_i(t)} >^2)_t^{1/2} (< \mathbf{r_j^2(t)} > - < \mathbf{r_j(t)} >^2)_t^{1/2}} \quad \text{(S1)}$$

where $r_i(t)$ and $r_j(t)$ denotes the vector of the ith and j^{th} C_α atom position as a function of time t. The quantity "$\mathbf{r_i(t)} - < \mathbf{r_i(t)} >$" corresponds to the fluctuation of the ith atom and "$\mathbf{r_j(t)} - < \mathbf{r_j(t)} >$" corresponds to the jth atom. For all the C_α atoms, a 326 $\times$ 326 correlation map was obtained for WT and pS44, using the 500 ns molecular dynamics simulation trajectory. The cross-correlation map was computed using the in-house script.

2 STRUCTURAL NETWORK ANALYSIS

Node centrality describes the distribution of the edges in the network. In protein networks, identifying the nodes with many edges (hubs) as well as segments with a high number of connections can give insight into the internal dynamics of protein regions. There are several different measures of node centrality, from the simplest one that counts the number of the edge of each node (degree) to the more complex such as (betweenness and closeness centralities). We already defined these centralities in the main text (see subsection 2.5).

The trajectories corresponding to the WT and pS44 were used to calculate the cross-correlation and network centrality. The cross-correlation calculation was explained in section 1.

We constructed a protein graph connectivity network from the cross-correlation matrix for WT and pS44. The α-carbon atom of each residue is considered to be a node in our network construction. The cross-correlation matrix elements are used to compute the weight of the edge. The weight corresponds to the probability of information transfer across the edge. Each edge in our network has an information transfer probability.

We calculated the node centralities for each residue. The centrality analysis provides a way to quantify the amount of information flow between nodes or edges of a network.

All the three centralities are computed using the Bio3d and igraph packages.

Table S1. The donor-acceptor distance between S44 and E335 for five systems. The second and third column list the average and standard deviations values.

System	Average dist ($\AA$)	Standard Deviation($\AA$)
Wild type(WT)	5.12	1.7
WT with Mg ions	7.2	1.9
Phosphorylated Serine (pS44)	10.89	1.854
pS44 with Mg ions	12.97	1.877

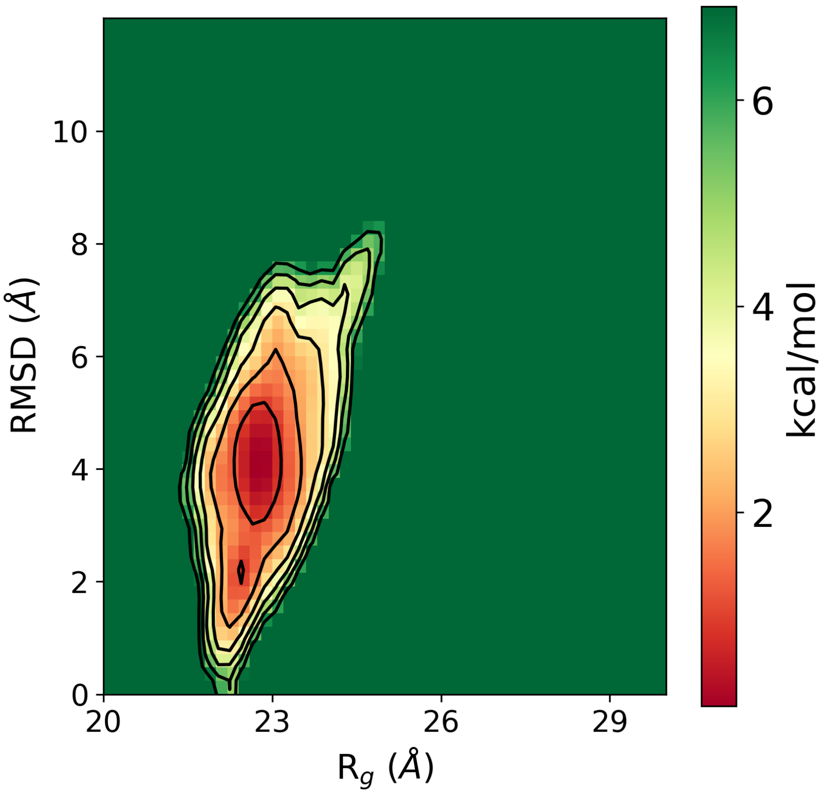

Figure S1. Free energy landscape of DNA polymrase β as a function of R_g (Å) and RMSD (Å) for WT in absence of Mg ions.

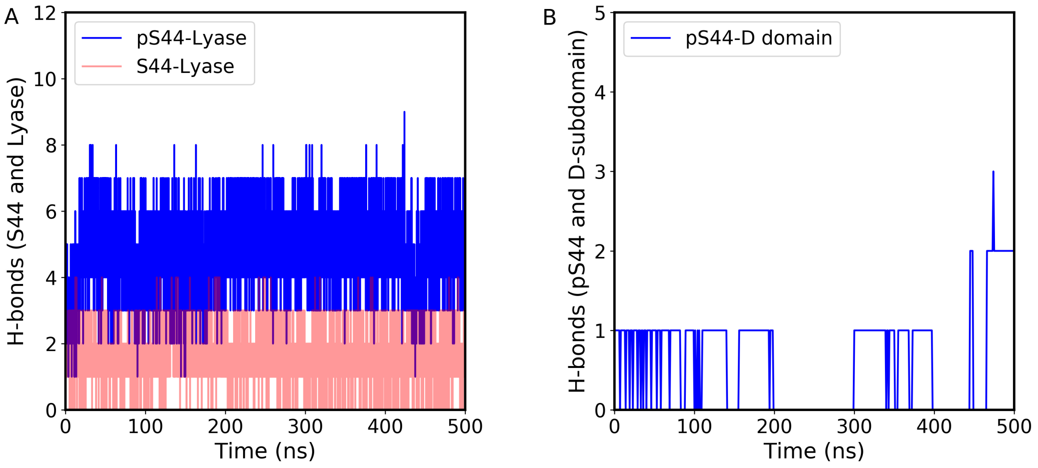

Figure S2. Time evolution of Hydrogen bonds (A) H-Bonds between Lyase domain and S44 for WT and pS44, (B)

Table S2. List of the salt bridges formed between the Mg binding sites and the different domains of DNA pol β.

System	Salt bridges
Wild type(WT)	D190-R149, D190-R254, D192-R258, D192-K234<br>D256-K234, D256-R254, D256-R258
WT with Mg ions	D190-R254, D254-R254, D256-R258
Phosphorylated Serine (pS44)	D190-R254, D192-R258, D256-K234<br>D256-R254, D256-R258
pS44 with Mg ions	D256-R254, D256-R258

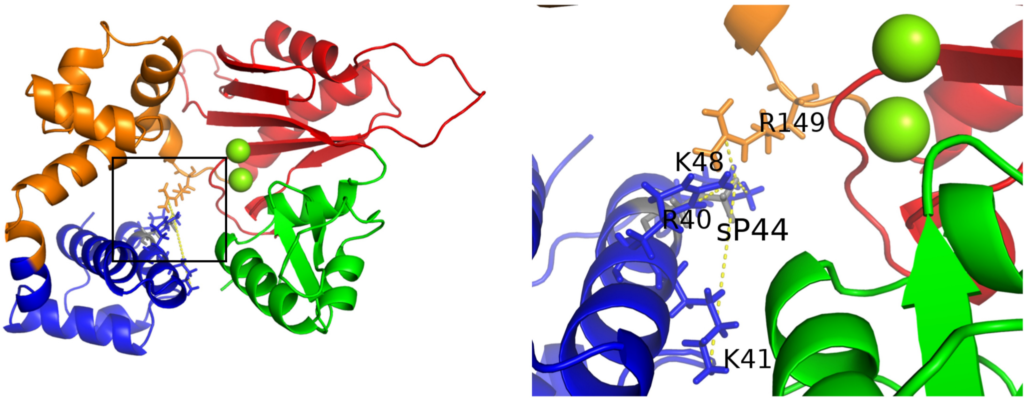

Figure S3. Schematic representation of salt -bridges. The inset shows the salt bridge formed by phosphorylated serine (pS44). The right panel shows the zoomed-up inset. Here, we show the salt bridge formed between pS44 and K41; K48; R40; and R149. The salt bridges formed between pS44 and K280; and R299 are shown in the main text.

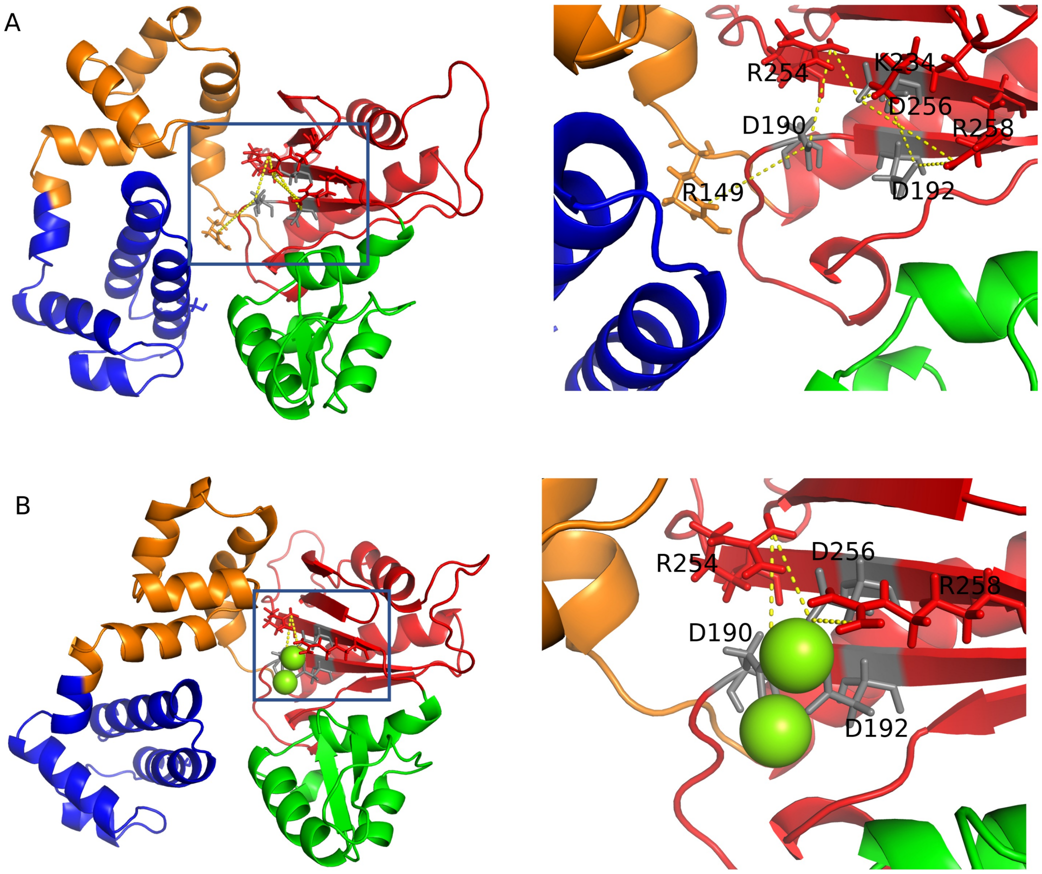

Figure S4. Salt bridges formed between the Mg binding sites (D190, D190, D256) and different domain and sub-domains shown on the DNA pol structure **(A)** Wild type (WT), **(B)** WT with Mg ions. The Mg ions are shown by sphere representation with green color. The Mg binding sites are shown in grey with stick representation.

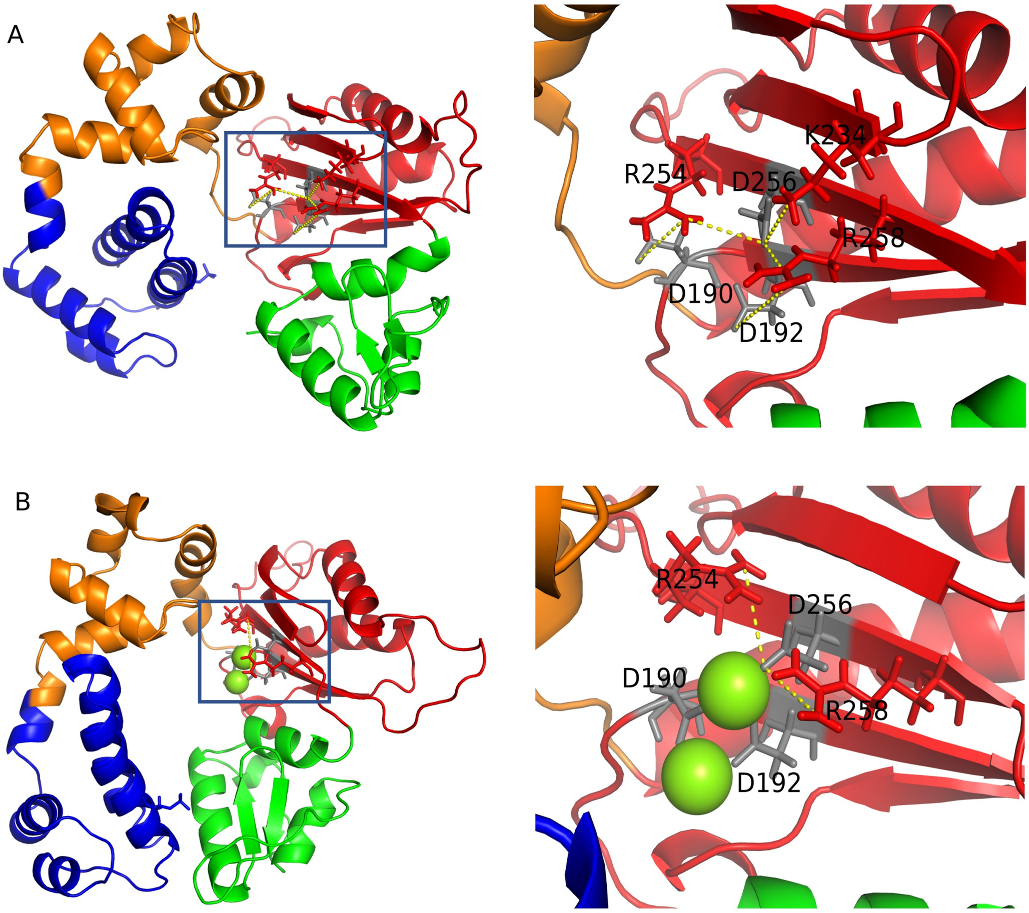

Figure S5. Salt bridges formed between the Mg binding sites (D190, D190, D256) and different domain and sub-domains shown on the DNA pol structure **(A)** Phosphorylated Serine (pS44), **(B)** pS44 with Mg ions. The Mg ions are shown by sphere representation with green color. The Mg binding sites are shown in grey with stick representation.

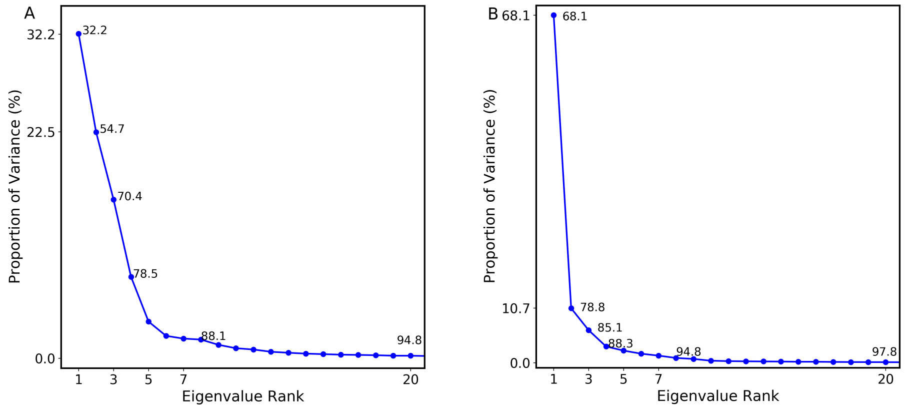

Figure S6. Scree plot for principal component analysis on the MD data of DNA pol β **(A)** WT, and **(B)** pS44.

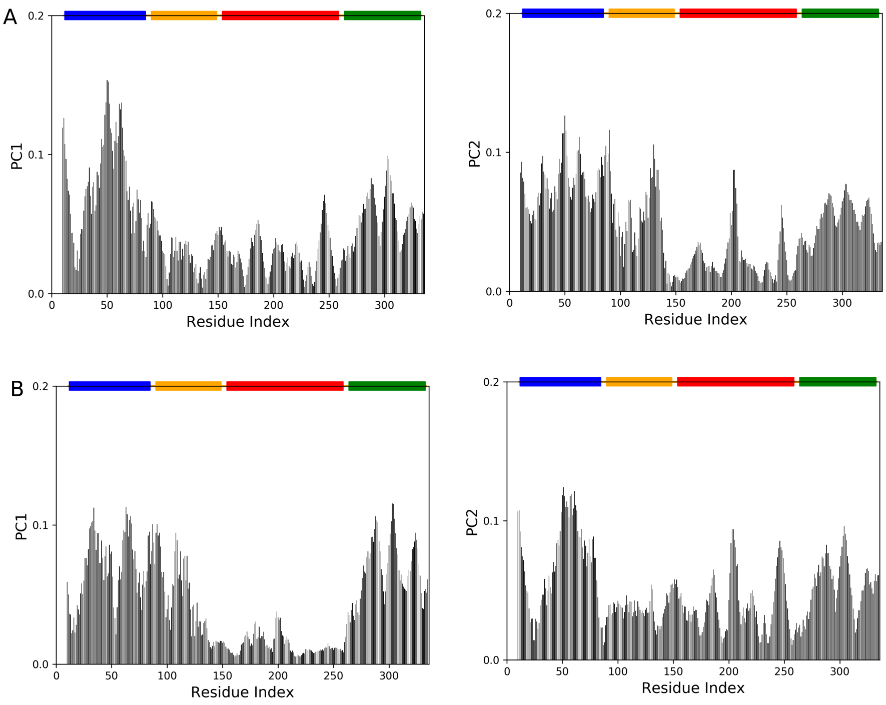

Figure S7. DNA polymerase β each residue contribution to principal component 1 (PC1) and principal component 2 (PC2) of **(A)** pS44, and **(B)**) WT.

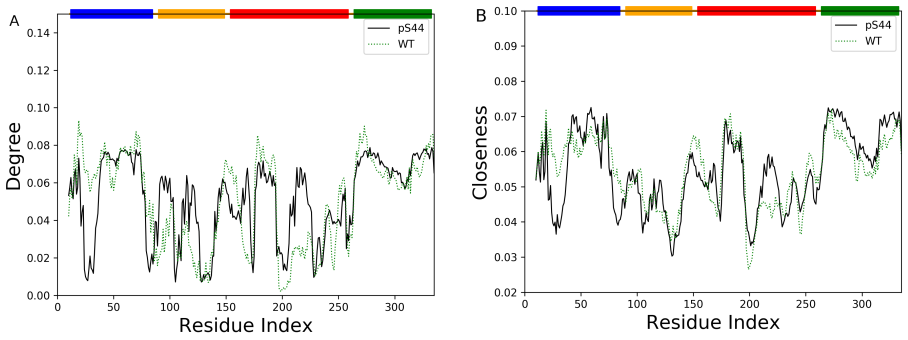

Figure S8. Network centrality analysis for WT and pS44 **(A)** Degree centrality for each residue of WT and pS44, and **(B)** Closeness centrality for each residue of WT and pS44. The rectangle shown in figure represents the different domains of DNA pol β.

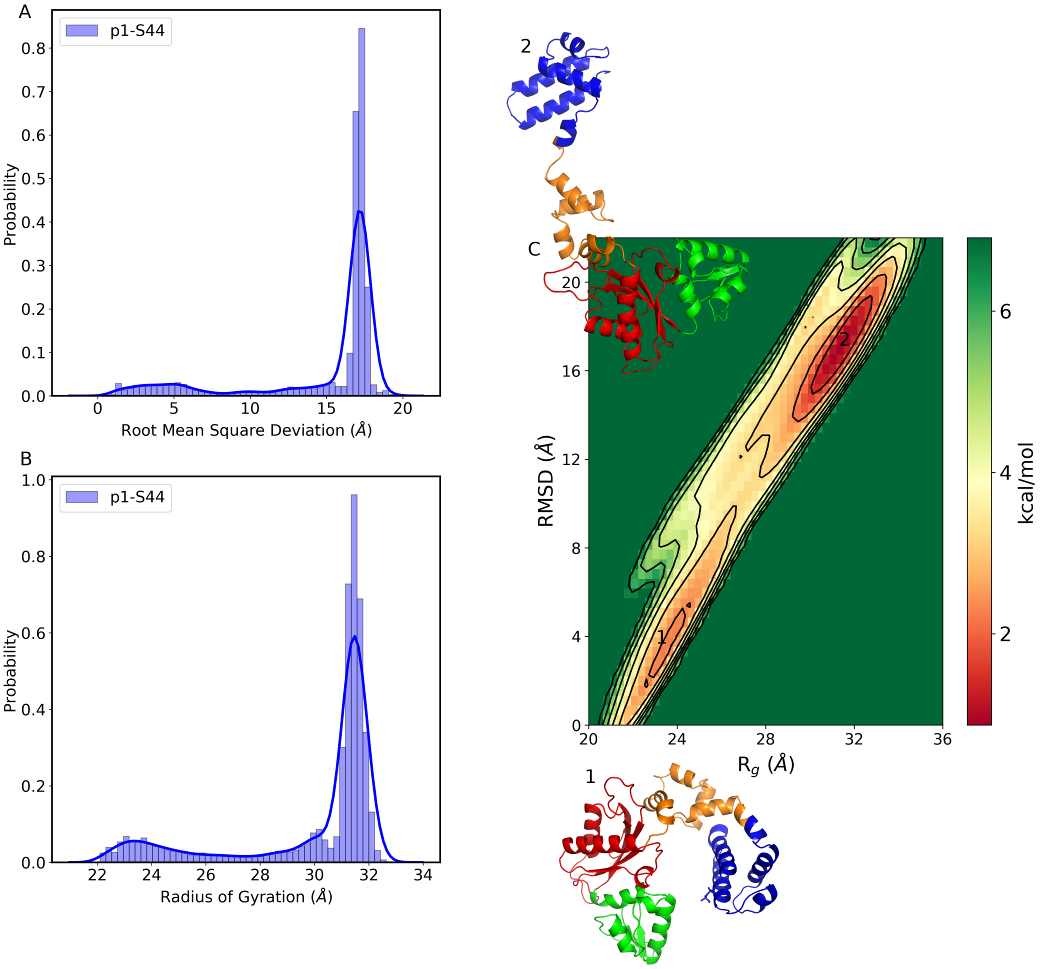

Figure S9. Probability distribution of **(A)** Root mean square deviation, and (B) radius of gyration for p1-S44. Free energy landscape of DNA pol β as a function of R_g and $RMSD$ for **(C)** p1-S44. Representative structure corresponds to minimum energy states are also shown.
